# Artificial intelligence to detect the femoral intertrochanteric fracture: The arrival of the intelligent-medicine era

**DOI:** 10.3389/fbioe.2022.927926

**Published:** 2022-09-06

**Authors:** Pengran Liu, Lin Lu, Yufei Chen, Tongtong Huo, Mingdi Xue, Honglin Wang, Ying Fang, Yi Xie, Mao Xie, Zhewei Ye

**Affiliations:** ^1^ Department of Orthopedics, Union Hospital, Tongji Medical College, Huazhong University of Science and Technology, Wuhan, China; ^2^ Department of Orthopedics, The Second Affiliated Hospital of Xiangya School of Medicine, Central South University, Changsha, China

**Keywords:** artificial intelligence, femoral intertrochanteric fracture, diagnosis, deep learning, convolutional neural network

## Abstract

**Objective:** To explore a new artificial intelligence (AI)-aided method to assist the clinical diagnosis of femoral intertrochanteric fracture (FIF), and further compare the performance with human level to confirm the effect and feasibility of the AI algorithm.

**Methods:** 700 X-rays of FIF were collected and labeled by two senior orthopedic physicians to set up the database, 643 for the training database and 57 for the test database. A Faster-RCNN algorithm was applied to be trained and detect the FIF on X-rays. The performance of the AI algorithm such as accuracy, sensitivity, miss diagnosis rate, specificity, misdiagnosis rate, and time consumption was calculated and compared with that of orthopedic attending physicians.

**Results:** Compared with orthopedic attending physicians, the Faster-RCNN algorithm performed better in accuracy (0.88 vs. 0.84 ± 0.04), specificity (0.87 vs. 0.71 ± 0.08), misdiagnosis rate (0.13 vs. 0.29 ± 0.08), and time consumption (5 min vs. 18.20 ± 1.92 min). As for the sensitivity and missed diagnosis rate, there was no statistical difference between the AI and orthopedic attending physicians (0.89 vs. 0.87 ± 0.03 and 0.11 vs. 0.13 ± 0.03).

**Conclusion:** The AI diagnostic algorithm is an available and effective method for the clinical diagnosis of FIF. It could serve as a satisfying clinical assistant for orthopedic physicians.

## Introduction

As the pivotal location of force conduction in the hip joint, the proximal femur could be damaged by the excessive violent load (Bhandari and Swiontkowski, 2017). Femoral intertrochanteric fracture (FIF) was the fracture of the proximal femur in the hip joint. It was a violent articular injury with a broad damage spectrum to the lower extremity motor system, which usually accompanied a high in-hospital death rate (6%–10%) and poor clinical outcome (Okike et al., 2020; Zhao et al., 2020). With the unsatisfied mortality, complications, mobility, and quality of life, FIF patients suffered from excruciating misery. After the injury of the hip joint, the initial diagnosis was commonly finished in the emergency department, and a conventional X-ray could be the primary diagnostic method to confirm whether a fracture occurred. Rather than other imaging modalities such as CT and MRI, X-ray was convenient, rapid, inexpensive, and easy to be recognized by radiologists or orthopedists. Generally, the ability to read X-ray images was an essential clinical skill that must be mastered by qualified doctors, which could guarantee accurate diagnosis and subsequent treatment. However, when it was under urgent situations in the emergency department (usually as the first visit for trauma patients) and lack of senior doctors, the probability of inducing the risk of missed diagnoses and misdiagnoses, especially for minor fractures, non-displaced fractures, or occult fractures increased significantly (Jamjoom and Davis, 2019). Several research studies had illustrated that missed diagnoses and misdiagnoses could even exceed 40% under severe and urgent conditions, which seriously affected the credibility of clinical diagnosis, delayed the launch of effective treatment, and induced poor clinical outcomes (Guly, 2001; Liu et al., 2021b). According to this, an accurate and credible auxiliary tool for bone fracture detection remained necessary.

With the advent of the intelligent-medicine era, a series of high technologies with great development had gradually been applied to the medical area to solve problems that were difficult to achieve in traditional medicine. For instance, with the supplementary mixed reality, the surgery of complex traumatic fractures could be performed easily (Liu P. et al., 2021), and with the assistance of a robot, the surgery could be performed more accurately and safely (Oussedik et al., 2020). With the enhancement of 5G communication technology, the telemedicine could be more realizable (Liu et al., 2020). As a representative technique of intelligent-medicine, artificial intelligence (AI) had also made great progress and become a powerful tool in medical image analysis with the application of machine learning (ML) and deep learning (DL) (Topol, 2019). AI was an interdisciplinary study of computer technology, mathematics, and cybernetics, which aimed to study, stimulate, and even surpass human intelligence. AI had formed several functional applications including 1) computer vision, 2) speech recognition, 3) natural language recognition, 4) decision planning, and 5) big data analysis. The primary advantage of AI was the ability to capture feature items of the target, which could be transformed into a performed method in the image analysis by AI. In previous research studies, AI had been applied to locate the abnormal area in the image of the pathological section, capsule endoscopy, ultrasound, and imageological examinations and achieved satisfying results in improving detection accuracy and diagnostic level (Ding et al., 2019; Nguyen et al., 2019; Norman et al., 2019; Wang et al., 2019; Chen et al., 2021). Therefore, in the present study, we first explored the ability of AI in FIF detection on X-ray images and then compared the difference between AI and orthopedic attending physicians. The result of this study could further verify the feasibility of AI-assisted medical diagnosis and provide a novel method for the clinical diagnosis of FIF.

## Methods

### Database and study design

As a multi-center study, the data of FIF X-rays were collected from five Chinese triple-A grade hospitals (Wuhan Union Hospital, Wuhan Puai Hospital, The Second Xiangya Hospital of Central South University, Xiangya Changde Hospital, and Northern Jiangsu People’s Hospital). The inclusion and exclusion criteria are shown in Table 1. A total of 700 X-rays from 459 FIF patients were acquired, including 459 FIF X-rays and 241 normal hip X-rays. Then, the acquired 700 X-rays were converted from Digital Imaging and Communications in Medicine (DICOM) files to Joint Picture Group (JPG) files with a matrix size of 600 × 800 pixels by Photoshop 20.0 (Adobe Corp., United States). These 700 JPG files were numbered using FreeRename 5.3 software (www.pc6.com). Through the random-number-table function in Excel (Microsoft Corp., United States), 700 JPG files were randomly divided into two datasets: a training database (including 643 files, consisting of 413 FIF and 230 normal hips, for AI learning and training) and the test dataset (including 57 files, consisting of 46 FIF and 11 normal hips, for effect validation). The ratio of the two datasets was nearly 9:1.

**TABLE 1:** Inclusion and exclusion criteria of data collection.

	Inclusion criteria	Exclusion criteria
1	Patients were adults (age >18 years old)	Juvenile patients (age <18 years old)
2	No other hip fractures were associated (such as the fractures of the femur, neck, femur head, and proximal femur that did not involve intertrochanteric areas)	Other hip fractures were associated
3	The preoperative anteroposterior X-ray was available and standard without any improper position, overexposure, ghosting, and shelters, such as plaster, splint, and metal objects on clothes	The preoperative anteroposterior X-ray was not performed in the hospital or no standard

**FIGURE 1:**
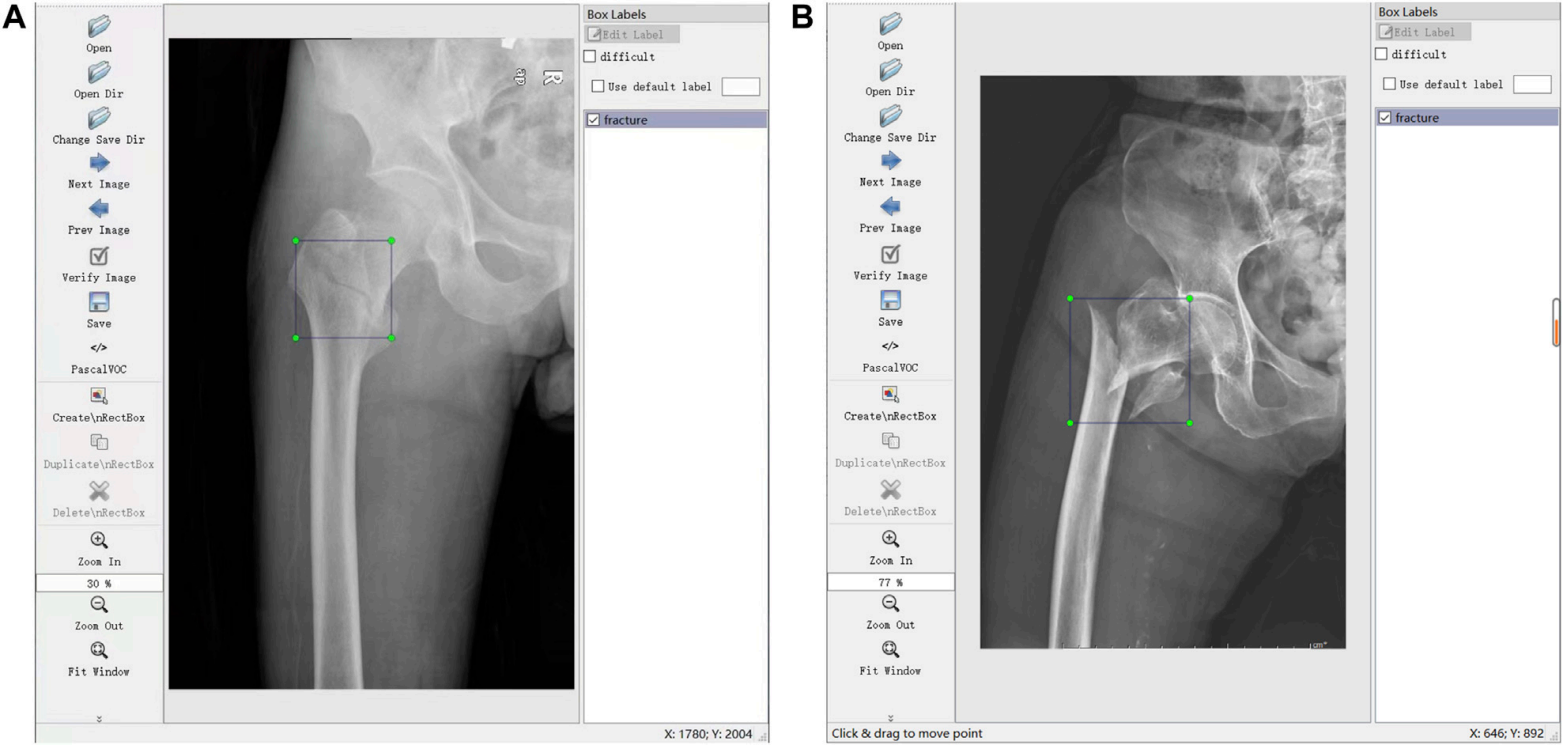
Illustrations of labeling methods. **(A)** Labeling of individual fracture lines. **(B)** Labeling of comminuted fractures.

Through the labeling software LabelImg (https://github.com/tzutalin/LabelImg), 700 JPG files were further confirmed and labeled with a tag of fracture (meant FIF) or normal hip for the subsequent training. Label works were performed (A and B) with more than 10 years of experience. Briefly, the files from the tab named [Open Dir] were imported, and then the label type “VOC label” (with the name suffix of “.xml”) from the tab named [Pascal VOC] was selected. The menu bar was opened by right-clicking the image, and the tab named [Create Rectangle] was selected to outline the individual fracture line (or fracture areas where fracture lines were not obvious, such as comminuted fractures) in the rectangle. Several standards of labeling needed to be stated. 1) The rectangle should cover target areas as small as possible to avoid leaving an invalid blank area. 2) If multiple target areas existed, they should be marked. 3) A certain range of errors was allowed, but ambiguous areas should not be marked. The illustration of the labeling method is shown in Figure 1.

Then, a type of AI recognition algorithm was designed and trained with the training database to learn the anatomical features of the hip on non-FIF X-rays and the characteristics of fracture lines on FIF X-rays. After the training process by training database, the algorithm could automatically recognize and label the suspicious area of FIF on X-rays in the test dataset, whose results could assist in FIF detection. Finally, to verify the difference between the algorithm and the human level, the recognized performance in the test dataset of the AI algorithm (in the form of accuracy, sensitivity, miss diagnosis rate, specificity, misdiagnosis rate, and time consumption) were compared with a panel of five orthopedic attending physicians (C, D, E, F, and G) in the emergency department of Wuhan Union Hospital. To protect patients’ privacy, all identifying information such as name, sex, age, and ID on the X-rays were anonymized and omitted when the data were first acquired. The study was approved by the Ethics Committee of Wuhan Union Hospital.

### Algorithm design, training, and performance assessment

**FIGURE 2:**
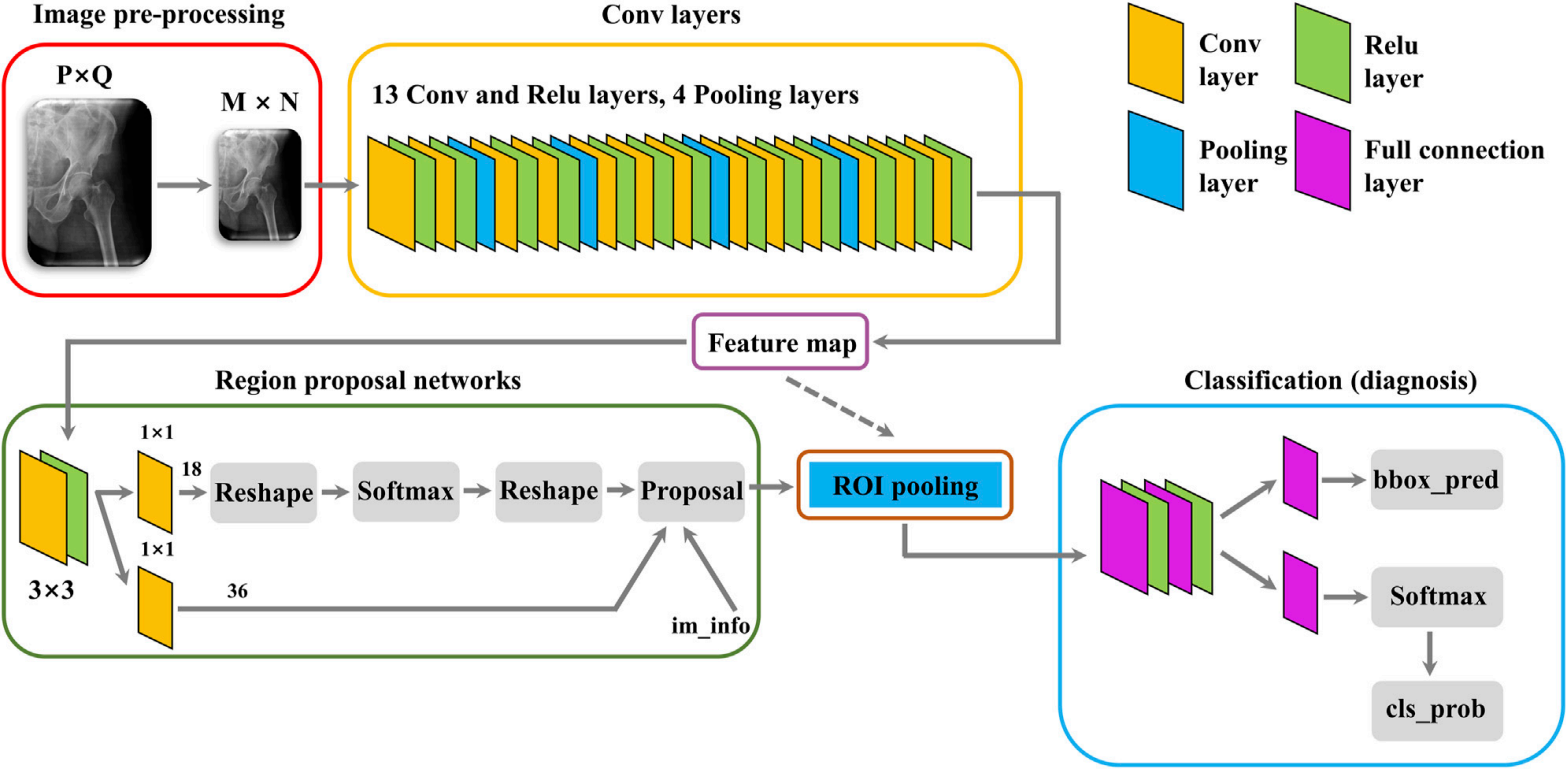
Structure of the Faster-RCNN algorithm.

A classical Faster-RCNN target detection algorithm was designed to recognize the fracture line of FIF X-ray (the structure of the algorithm was shown in Figure 2). Briefly, after the establishment of the Faster-RCNN algorithm, the training database was first enhanced by the algorithm including image rollover, rotation, cropping, and blurring, which multiplied the original training database fivefold (643 files to 3,215 files). Then, the amplified training database was imported into Faster-RCNN for algorithm training. During the training process, images were scaled and preprocessed by the algorithm and then input into the convolutional neural network (CNN) for image feature extraction. The extracted feature map was fed into the Region Proposal Network (RPN) to generate an “anchor frame”, and then the full connection layer was used to make a preliminary decision on the “anchor frame”. The obtained preliminary decision and previously obtained features were sent into the region of interest (ROI) pooling layer to fix input dimensions, and finally, the prediction results were obtained by box regression classification.

After Faster-RCNN training, the test database was imported to verify the training effect, the performance of the algorithm to recognize FIF or normal hip was calculated according to outputted results (the FIF image with a red frame, normal hip with non) and the real diagnosis (based on the labeling of physician A and B). Finally, the performance of Faster-RCNN was expressed in the form of accuracy, sensitivity, miss diagnosis rate, specificity, misdiagnosis rate, and time consumption.

### Performance assessment of orthopedic attending physicians

To assess the diagnostic performance of orthopedic physicians on the clinical front line, in this study a panel of five orthopedic attending physicians was recruited from the emergency department of Wuhan Union Hospital. All of them had experienced the emergency management of traumatic fractures and possessed a professional ability to read X-ray images. The panel of orthopedic attending physicians was independent of this study and did not participate in any processes of this project. They were informed to diagnose the test dataset independently as FIF or normal hip without any reminder. During the whole process, conversation and consultation were forbidden and the time was unlimited. Then, their diagnostic results were collected and judged according to the real diagnosis. The accuracy, sensitivity, miss diagnosis rate, specificity, misdiagnosis rate, and time consumption were calculated as the performance.

Finally, the performance of AI and orthopedic attending physicians were compared in order to evaluate the diagnostic ability and clinical feasibility of the algorithm.

### Statistics

The data of this study were presented as the mean ± standard deviation (SD) or percentage, and the statistical analysis was performed using GraphPad Prism 7.0 software (GraphPad Corp., United States). The significance between the algorithm and orthopedic attending physicians was evaluated by the Student’s t-test. *p* < 0.05 was considered to indicate statistical significance.

## Results

### Performance of the algorithm

**FIGURE 3:**
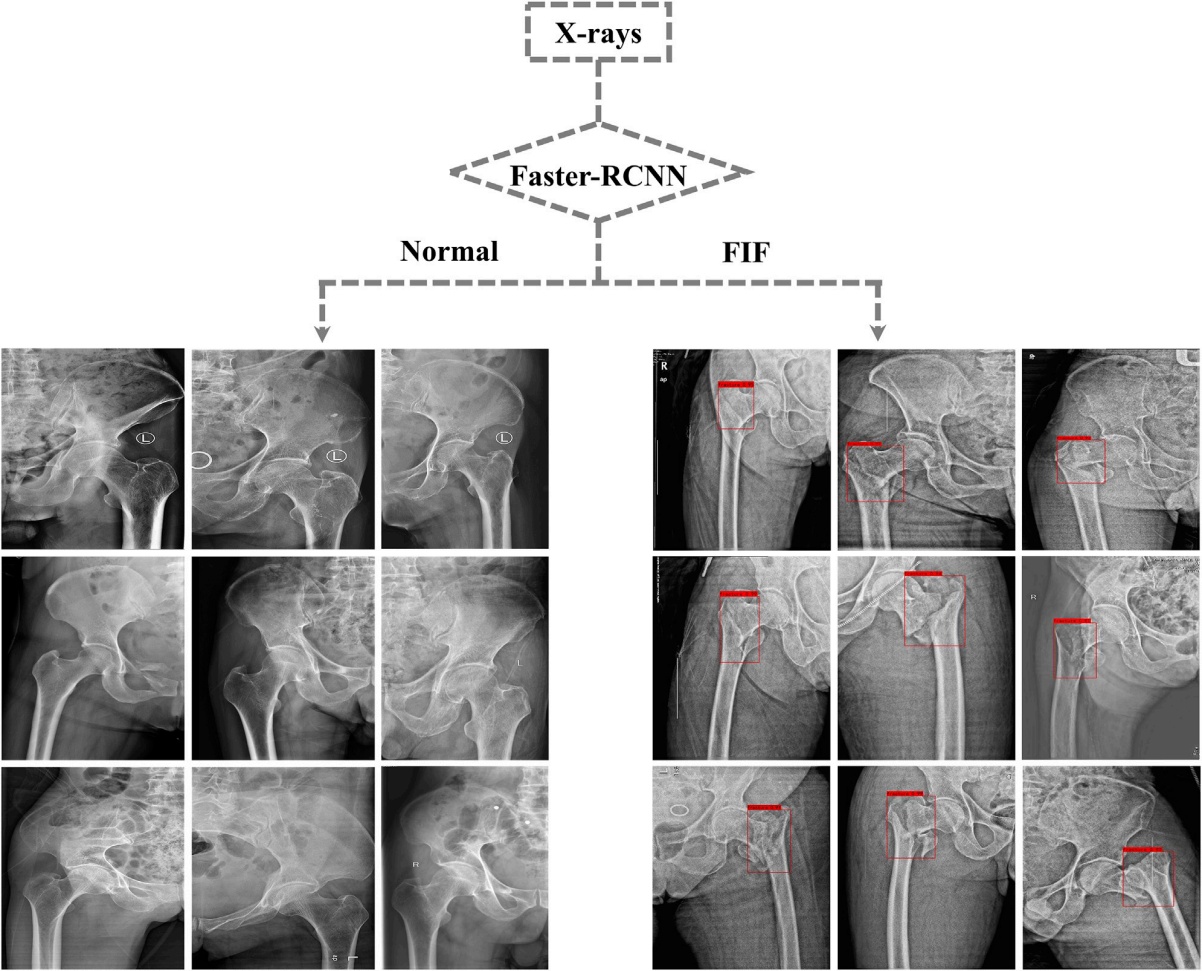
Part of output X-rays from the test dataset. Suspicious fractures were labeled with a red rectangle by the Faster-RCNN algorithm.

After training, the algorithm gave the test database a diagnosis according to features learned before. If the diagnosis was FIF, there would be a red rectangle on the suspicious fracture line (As shown in Figure 3). 1) The F1 score (an indicator used to measure the accuracy of the dichotomous model in statistics. It took into account both the accuracy and recall of classification models. The F1 score could be regarded as a harmonic average of model accuracy and recall with a maximum value of 1 and a minimum value of 0), 2) recall (the ratio of the amount of relevant information checked out from the database to the total amount), 3) precision, 4) AP (average precision), mAP (mean average precision) and IOU (intersection over union), 5) AUC (area under the curve) and ROC (the receiver operator characteristic curve), and 6) accuracy, sensitivity, missed diagnosis rate, specificity, and misdiagnosis rate were used to evaluate the effect and performance of the algorithm. The F1 score, recall, precision, AP, mAP, IOU, AUC, and ROC are shown in Supplementary Material. The accuracy (0.88), sensitivity (0.89), missed diagnosis rate (0.11), specificity (0.87), and misdiagnosis rate (0.13) were calculated and exported by the algorithm.

### Performance of orthopedic attending physicians

The diagnostic results of five orthopedic attending physicians were collected to calculate the accuracy, sensitivity, missed diagnosis rate, specificity as well as misdiagnosis rate, and the time consumption of each one was also recorded. The data are shown in Table 2.

**TABLE 2:** Performance of orthopedic attending physicians.

Performance	Physician C		Physician D		Physician E		Physician F		Physician G	
Total correct/incorrect	47/10		49/8		47/10		46/11		51/6	
Diagnostic result	FIF	Non	FIF	Non	FIF	Non	FIF	Non	FIF	Non
FIF (real fracture)	40	6	41	5	39	7	39	7	42	4
Non (real normal hip)	4	7	3	8	3	8	4	7	2	9
Accuracy	0.82		0.86		0.82		0.81		0.89	
Sensitivity	0.87		0.89		0.85		0.85		0.91	
Missed diagnosis rate	0.13		0.11		0.15		0.15		0.09	
Specificity	0.64		0.73		0.73		0.64		0.82	
Misdiagnosis rate	0.36		0.27		0.27		0.36		0.18	
Time consumption (min)	16 min		17 min		19 min		21 min		18 min	

### The comparison of the algorithm and orthopedic attending physicians

**FIGURE 4:**
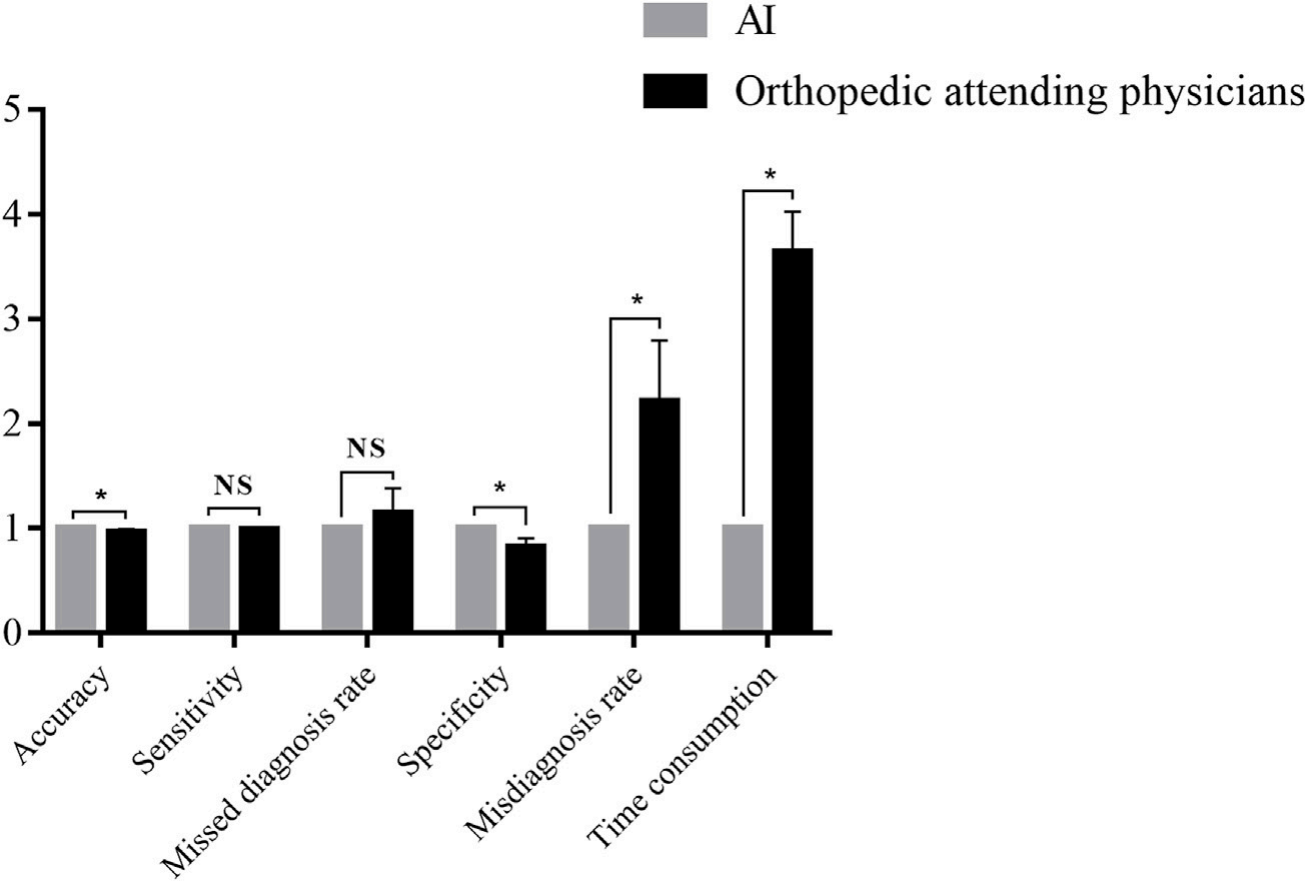
Comparison between the Faster-RCNN and orthopedic attending physicians. *NS: not significant. *p < 0.05.

The performance of Faster-RCNN and orthopedic attending physicians were compared. The results are shown in Table 3 and Figure 4.

**TABLE 3:** Comparison between the Faster-RCNN and orthopedic attending physicians.

Performance	Algorithm	Orthopedic attending physician	T value	*p* value
Accuracy	0.88	0.84 ± 0.04	2.64	0.03
Sensitivity	0.89	0.87 ± 0.03	1.37	0.21
Missed diagnosis rate	0.11	0.13 ± 0.03	1.37	0.21
Specificity	0.87	0.71 ± 0.08	4.69	0.002
Misdiagnosis rate	0.13	0.29 ± 0.08	4.69	0.002
Time consumption	5 min	18.20 ± 1.92 min	15.34	<0.001

In the diagnosis of the test database, the results illustrated an accuracy of 0.88 for Faster-RCNN vs. 0.84 ± 0.04 for orthopedic attending physicians (*p* < 0.05), a sensitivity of 0.89 for Faster-RCNN vs. 0.87 ± 0.03 for orthopedic attending physicians (*p* > 0.05), a missed diagnosis rate of 0.11 for Faster-RCNN vs. 0.13 ± 0.03 for orthopedic attending physicians (*p* > 0.05), a specificity of 0.87 for Faster-RCNN vs. 0.71 ± 0.08 for orthopedic attending physicians (*p* < 0.05), a misdiagnosis rate of 0.13 for Faster-RCNN vs. 0.29 ± 0.08 for orthopedic attending physicians (*p* < 0.05) and a time consumption of 5 min for Faster-RCNN vs. 18.20 ± 1.92 min for orthopedic attending physicians (*p* < 0.05).

## Discussion

FIF was a kind of high-energy injury with complex complications in the hip joint, which seriously threatened the health of patients and even led to death (Simunovic et al., 2010; Katsoulis et al., 2017). The early diagnosis and surgical treatment were defined as critical factors in reducing postoperative complications and mortality (Khan et al., 2013; Colais et al., 2015; Investigators, 2020). However, under emergencies the clinical diagnosis of FIF was often unsatisfactory due to an unclear X-ray presentation, especially in minor fracture, non-displaced fracture, or occult fracture, which could finally lead to missed diagnoses or misdiagnoses. A retrospective analysis in the emergency department illustrated that missed diagnoses and misdiagnoses mostly occurred to the hip fracture (37.3%) rather than other limb fractures, and doctors were more likely to make mistakes between 5 p.m. and 3 a.m. due to fatigue and other factors. After diagnosis correcting, there were more than 55% of patients who still required further treatment such as cast immobilization or even surgery (Mattijssen-Horstink et al., 2020). Hence, there was an urgent demand to find an auxiliary tool to assist clinical fracture diagnosis.

In this study, an AI algorithm (Faster-RCNN) was applied to help doctors automatically diagnose the clinical FIF on hip X-rays, which had shown a satisfying performance compared with five orthopedic attending physicians. After feature extraction and learning from the training database of 643 X-rays, the test database of 57 X-rays was imported to confirm the effect of the algorithm. From results in this study, Faster-RCNN showed an excellent ability. Compared with the performance of orthopedic attending physicians, Faster-RCNN performed better in accuracy (0.88 vs. 0.84 ± 0.04), specificity (0.87 vs. 0.71 ± 0.08), and especially time consumption (5 min vs. 18.20 ± 1.92 min), with a nearly fourfold increase. It meant Faster-RCNN performed better in the total recognition of FIF from the normal hip and got a lower misdiagnosis rate than the human level (0.13 vs. 0.29 ± 0.08). Strikingly, as for the time consumption, Faster-RCNN was drastically faster than the manual diagnostic speed. Despite the sensitivity and missed diagnosis rate of Faster-RCNN were not significantly improved than the human level, it still reached the level of orthopedic attending physicians (0.89 vs. 0.87 ± 0.03) and (0.11vs. 0.13 ± 0.03). Also, the performance of orthopedic attending physicians in this study proved the conclusion of Mattijssen’s research (Mattijssen-Horstink et al., 2020). Missed diagnoses and misdiagnoses really existed in the daily medical work and posed threat to the diagnosis, treatment, and rehabilitation of patients, which still kept demanding for a clinical auxiliary tool, such as AI. The performance of AI in this study showed that after training, AI could already serve as an assistant for doctors in FIF diagnosis and even perform better than the human level in some aspects. Moreover, according to the nonemergency and time-free testing environment in the process of human performance assessment, which did not simulate real urgent circumstances very well, the authors believed the performance of AI could be even better than the reality of the emergency department.

There were already studies indicating a satisfying performance of AI in the assistance for clinical disease diagnosis. For example, in the diagnosis of lung disease, Yoo designed an AI model through chest X-ray analysis of 5,485 smokers. Through training, the sensitivity and specificity of the model reached 0.86 and 0.85 in lung nodules automatic recognition on X-ray, 0.75 and 0.83 of lung cancer recognition with a 0.38 positive predictive value and a 0.99 negative predictive value, which were more accurate than radiologists (Yoo et al., 2020). During the period of coronavirus disease in 2019 (COVID-19), Wang established a CNNs algorithm to learn chest CT of 1,647 COVID-19 infected patients and 800 noninfected patients from Wuhan, China. Diagnostic tests were carried out by suspected infected patients in multiple clinical institutions, and the sensitivity and specificity of the model reached 0.92 and 0.85. The median time consumption was 0.55 min, which got 15 min less than that of the manual level and provided great help for rapid diagnosis in the fight against the epidemic (Wang et al., 2020). Zhao measured the size of tumors in the lungs, liver, and lymph nodes of patients based on CT with different slice intervals, and the intra- and inter-reader variability were also analyzed by linear mixed-effects models and the Bland-Altman method (Zhao et al., 2013). Research studies meant a well-trained AI algorithm was fully competent for the imaging diagnosis of clinical diseases and had reached the level of imaging physicians, which effectively sped up the workflow of imaging interpretation. Also, there were various research studies that implemented the automated diagnosis of orthopedic diseases with the AI algorithm. For instance, Gan trained CNN with 2,340 anterior-posterior wrist radiographs, and finally, the algorithm got an accuracy of 0.93, sensitivity of 0.9, and specificity of 0.96 in the diagnosis of distal radius fractures, which showed a similar ability to orthopedists and radiologists (Gan et al., 2019). Choi established a dual-input CNN upon 1,266 pairs of anteroposterior or lateral elbow radiographs for the automated detection of supracondylar fracture, after the DL process, the algorithm expressed a specificity of 0.92 and a sensitivity of 0.93, which provided a comparable diagnostic ability to radiologists (Choi et al., 2020). As for proximal humeral fractures, Chung set up a deep CNN and trained the algorithm with 1,891 X-rays of the shoulder joint (515 normal shoulders, 346 greater tuberosity fractures, 514 surgical neck fractures, 269 three-part fractures, and 247 four-part fractures). The algorithm showed excellent performance with 0.96 accuracy, 0.99 sensitivity, and 0.97 specificity for distinguishing normal shoulders from proximal humerus fractures. Moreover, in the fracture type classifying, the trained algorithm also showed promising results with 0.65–0.86 accuracy, 0.88–0.97 sensitivity, and 0.83–0.94 specificity, which performed better than general physicians and similar to orthopedists specialized in the shoulder (Chung et al., 2018). The scaphoid fracture was the most common carpal bone fracture, whose diagnosis might be difficult, particularly for physicians inexperienced in hand surgery, to accurately evaluate and interpret wrist radiographs due to its complex anatomical structures. In terms of this issue, Ozkaya built CNN to detect scaphoid fractures on anteroposterior wrist radiographs and also compared the performance of the algorithm and doctors in the emergency department. This study included a total of 390 patients with AP wrist radiographs, and the algorithm expressed a 0.76 sensitivity and 0.92 specificity in identifying scaphoid fractures, which showed that CNN’s performance was similar to a less experienced orthopedic specialist but better than the physician in the emergency department (Ozkaya et al., 2022). In addition to fractures, AI also improved the interpretation of the skeletal age and the intelligent diagnosis of scoliosis, osteosarcoma, osteoarthritis, motor system injury as well as other orthopedic diseases (Watanabe et al., 2019; Garwood et al., 2020; Gorelik and Gyftopoulos, 2021). All of research studies confirmed the feasibility and value of AI in clinical diagnosis (the summary of the performance of AI are shown in Table 4).

**TABLE 4:** Summary of the performance of AI in fracture diagnosis.

Fracture diagnosis	Database size	Accuracy	Sensitivity	Specificity	Reference
Distal radius fractures	2,340	0.93	0.9	0.96	Gan et al. (2019)
Supracondylar fractures	1,266	—	0.93	0.92	Choi et al. (2020)
Proximal humeral fractures	1,891	0.96	0.99	0.97	Chung et al. (2018)
Scaphoid fractures	390	—	0.76	0.92	Ozkaya et al. (2022)
Femoral intertrochanteric fractures (this study)	700	0.88	0.89	0.87	—

Compared with other similar studies, the innovative algorithm Faster-RCNN was constructed at the beginning of the present study and first applied in the detection of FIF. As the first strength of the study, Faster-RCNN was the further evolution of R-CNN and Fast R-CNN with a superior performance, which greatly improved the accuracy and speed of detection. The end-to-end target detection framework was also truly realized. In principle, Faster R-CNN could be regarded as a system combined with RPN modules of Region Generation Network on the basis of Fast R-CNN. The role of selective search in the Fast R-CNN system was replaced by the Region Generation Network. And the core concept of RPN was applying CNN to generate the Region Proposal Network directly with the essence of the sliding window. As a result, it increased the speed of suggestion box generation to an average of 10 ms. From the data of this present study, the performance of Faster-RCNN was satisfying in the fracture diagnosis. Although, some of the performances were not as high as compared with other studies, we considered that differences might be caused by different anatomical features, the size of the database, and the diversity of labeling methods. Even so, the results of the study still indicated the feasibility and necessity of AI assistance in the diagnosis of FIF. The second strength of the present study was the comparatively small database, which might also be a limitation. Researchers in the field of AI-aided medicine always excessively pursued the larger database. Definitely, we believed that a high-volume database was the guarantee for the well-training of the AI algorithm. However, the satisfying result of this study also meant the mature algorithm structure and the accurate and effective labeling method were also equally important, which determined ultimate effectiveness. Third, as a multi-center study of five Chinese triple-A grade hospitals, the multiformity of our data further ensured the compatibility, applicability, and maturity of the well-trained algorithm. The performance of the model verified in different data environments would be more credible.

Through the research of AI application in medicine, we confirmed that AI had brought clinical work with obvious benefits. 1) Removing the workload of clinicians. The most striking feature of AI was the ability of automation. In the traditional model, the work of X-ray diagnosis was a laborious and labor-consuming process, whose efficiency always declined with the increasing workload. AI could process and recognize X-rays efficiently without fatigue, which simplified the workflow and reduced the difficulty (Bi et al., 2019). 2) Decreasing the occurrence of misdiagnoses and missed diagnoses. Diagnostic mistakes were inevitable during the busy medical work. After training with the labeling from veteran senior doctors, the algorithm could be recognized as a medical assistant with extensive experiments. Also, with the peculiar ability of image features extracting and suspicious signs locating, the screening level of the algorithm was beyond that of visual observation of humans. Hence, the AI-generated diagnosis could be a powerful reference for clinical doctors to avoid a large portion of diagnostic errors. 3) Accelerating the generation of clinical diagnosis. The automated diagnosis of AI could be generated in several seconds, which was more effective and stable rather than that of humans. 4) Improving diagnostic reliability. 5) Providing more guarantee to patient health (Liu et al., 2021a). 6) Saving medical resources and promoting rational redistribution. Medical resources were unevenly distributed whether between the emergency department and other medical departments or between underdeveloped regions and developed regions. With the promotion of AI in medicine, limited medical resources could be saved for redistribution. The predicament of medical deficiency in the emergency department or in remote and backward areas would also be alleviated. 7) Improving the clinical ability of physicians. In addition to providing reference to avoid medical negligence, intelligent diagnosis from AI could also provide a detailed and quantitative analysis rather than a qualitative conclusion, which would expand the limited clinical knowledge of junior doctors and improve their clinical diagnosis level. The physician’s continuous studying and progress in the working environment would also be achieved (Gao et al., 2022). In the future, the well-trained AI model could also be embedded in the picture archiving and communication (PACS) system to realize real-time and more efficient guidance, which could avoid the tedious work of image acquisition and model importing. We believed these benefits must be a great enhancement for clinical work.

However, according to possible computational errors and potential medical risks brought by an incomplete algorithm, the AI-generated diagnosis was best used only as a reference for doctors, which proposed that we should apply AI as clinical assistance, rather than a replacement. The final diagnosis still required supervision from a senior physician with extensive experience.

There were also some limitations of this study: 1) the database was not large enough, which would influence the final performance of the AI algorithm; 2) the database of this study only consisted of anteroposterior hip X-ray, which was not suitable for the diagnosis of lateral film; 3) the whole study merely focused at the identification of FIF and the fracture classification was uninvolved. Fracture classification was also important to determine the therapeutical principle and surgical plan, which would be more convenient and functional if it could be realized by the AI diagnosis. 4) The external validation dataset was not set in the verification process. The external validation dataset was an independent dataset and unknown to the algorithm, which could verify the performance of transportability and generalization of the algorithm temporally and geographically. According to the limited data in this stage of our study, to guarantee the training effect of the algorithm in priority, the setting of an external validation dataset was not available. In future research, the aforementioned points would be further improved.

## Conclusion

The AI diagnostic algorithm is an available and effective method for the clinical diagnosis of FIF, which could serve as a satisfying clinical assistant for orthopedic physicians.

## Data Availability

The original contributions presented in the study are included in the article/supplementary material. Further inquiries can be directed to the corresponding authors.
